# Developing a specific model to exploring the determinant of individuals’ attitude toward forest conservation

**DOI:** 10.3389/fpsyg.2024.1481087

**Published:** 2024-10-18

**Authors:** Rahim Maleknia, Reza Azizi, Aureliu Florin Hălălișan

**Affiliations:** ^1^Department of Forestry, Lorestan University, Khorramabad, Iran; ^2^Department of Forestry, Behbahan Khatam Alanbia University of Technology, Behbahan, Iran; ^3^Associate professor, Department of Forest Engineering, Faculty of Silviculture and Forest Engineering, Transilvania University of Brasov, Brașov, Romania

**Keywords:** participatory forest conservation, environmental awareness, environmental responsibility, biospheric values, Zagros forests

## Abstract

**Introduction:**

In the context of forest conservation, individuals’ attitude can significantly influence their intentions and subsequent behaviors towards conserving forests. However, there is a research gap when it comes to understanding the determinants of individuals’ attitude towards forests conservation. To address this gap, the present study aimed to investigate the influence of variables associated with values from value-belief-norm theory, awareness of consequences, and ascription of responsibility from norm activation model on individuals’ attitudes as main determinant of behavior intention.

**Methods:**

The Data of study was collected from a sample of 200 students from Behbahan University, Iran. through a questionnaire designed specifically for this purpose. The validity and reliability of questionnaire were confirmed. Structural equation modeling was employed to analyze the collected data.

**Results:**

The findings of the study revealed that the examined variables were able to describe 88.6% of the variance in individuals’ attitude towards forest conservation. Specifically, it was found that biospheric (ƛ= 0.097) and altruistic (ƛ= 0.385) values, ascription of responsibility (ƛ= 0.150), and awareness of consequences (ƛ= 0.380) had significant and positive effects on individuals’ attitude towards forest conservation. On the other hand, egoistic values exhibited a significant and negative impact (ƛ= –0.071) on individuals’ attitude.

**Discussion:**

These research findings hold significant implications for planners and policymakers involved in forest conservation efforts. By understanding the factors that shape individuals’ conservation attitudes, decision-makers can develop targeted strategies and interventions to strengthen positive attitudes towards forest conservation. Given the positive influence of biospheric values and awareness of consequences, developing awareness-raising programs to enhance individuals’ environmental knowledge and the awareness of outcomes of their conservation actions can be considered as strategy to strengthen public’s attitude and improvement their participation in forest conservation projects.

## 1 Introduction

Forests represent invaluable natural resources that provide a wide array of ecosystem services, social benefits, and economic contributions to humanity. In the contemporary context, the emergence of critical phenomena such as climate change (Jama et al., 2023; Liu et al., 2023; Maleknia and Salehi, 2024; Roy and Bhan, 2024), the conservation of water and soil resources (Geng et al., 2024; Rhodes et al., 2018), ensuring food security (Khosravi et al., 2016), and the significance of forests in supporting local communities and livelihoods (Bazgir et al., 2024; Khosravi et al., 2014) has underscored the heightened importance of forests for both individuals and governing bodies. Consequently, the imperative to forests conservation has become more pronounced than ever before. The destruction of forests is increasingly driven by factors such as population growth, the livelihoods of local communities, and shifting consumption patterns (Delpasand et al., 2022a; Gulzar et al., 2024; Mansori et al., 2023). This trend poses significant local and global consequences, including climate change and related events. Therefore, to mitigate these risks and to preserve the essential role of forests in biodiversity conservation, the development of forest conservation programs is imperative (Börner et al., 2020). Despite the considerable importance placed on forest conservation, forest degradation and deforestation continues to transpire across various regions globally, predominantly driven by anthropogenic factors (Barabadi et al., 2020; Cabral et al., 2024; Delpasand et al., 2022b; Lacan et al., 2024; Savari et al., 2020). These deforestation events engender multifaceted damages, including the exacerbation of climate change, thereby yielding ramifications that extend beyond regional boundaries and impact the entire globe (Cortner et al., 2024; Mansori et al., 2023). Nevertheless, owing to the vast expanse of forested areas, fiscal constraints, and limitations in available personnel, governments alone remain insufficient in their capacity to safeguard these invaluable ecosystems (Maleknia and ChamCham, 2024). Consequently, the achievement of this goal necessitates the active involvement and collaborative efforts of a diverse array of stakeholders, spanning both groups and individuals, working collectively toward this common goal (Sattayapanich et al., 2022; Zhang, 2022).

Engaging the public in forest conservation efforts presents a complex challenge, largely due to the diverse stakeholders involved, each with varying interests, and the existing distrust between governmental institutions and the public.(Khedrizadeh et al., 2017; Sapkota et al., 2020; Smith et al., 2024). Individuals’ attitude and behaviors play a crucial role in overcoming these challenges, as individual actions directly impact the effectiveness of conservation initiatives. Environmental psychology offers valuable insights into understanding these attitudes and behaviors by examining how individuals perceive and interact with natural environments. In the context of forest conservation, it highlights the importance of fostering voluntary participation (Savari et al., 2020). When people choose to engage in conservation efforts, such as reforestation or reducing resource consumption, they actively contribute to the preservation of forest ecosystems. Voluntary actions are particularly important, as they reflect a personal commitment to sustainability, helping bridge the gap between public mistrust and the need for collective action (Khedrizadeh et al., 2017). By leveraging environmental psychology, more effective strategies can be developed to inspire public participation and ensure long-term forest conservation. Environmental psychology is a branch of psychology that examines the interplay between individuals and their environments, which can be both natural and built (Fang et al., 2023). This field investigates how various environmental factors influence human cognition, emotion, and behavior, as well as how human actions impact these environments (Ruotolo et al., 2024).

Psychological models such as the Theory of Planned Behavior (TPB) (Maleknia and ChamCham, 2024; Savari et al., 2022a; Savari and Khaleghi, 2023; Sun et al., 2022), the Values-Beliefs-Norms (VBN) Model (Hong et al., 2024; Mohammadi et al., 2024b), and Social Cognitive Theory (SCT) (Savari et al., 2022b; Yazdanpanah et al., 2015), health belief model (Ataei et al., 2021; Boazar et al., 2020; Nasiri et al., 2024; Shahangian et al., 2022), theory of interpersonal behavior (Kim et al., 2016; Sung et al., 2019), have been employed to explore the complexities of individuals intentions and behaviors regarding environmental behaviors and forest conservation. In these models, the primary focus has been on determining the variance in individuals’ intentions and behaviors and influencing factors which shape these variables (Maleknia and Salehi, 2024; Mohammadi et al., 2024a). In forest conservation field, psychological models such as Norm Activation Model (NAM) (Latifinia et al., 2022; Savari et al., 2023), TPB (Maleknia and ChamCham, 2024; Savari and Khaleghi, 2023) have been applied. In these research, various behavioral variables such as attitude have been examined within these frameworks. In psychology, attitude is considered a crucial determinant of behavior, as it influence individuals’ intentions directly, which can ultimately lead to actual behavior (Ajzen, 1993), with its influence on intention formation in the context of forest management being well-documented (Chamcham et al., 2024; Maleknia and ChamCham, 2024; Savari and Khaleghi, 2023). Despite the importance of examining individuals’ attitude in shaping their behavioral intentions and actual behavior in the context of forest conservation, there exists a research gap in this area. In field of forest conservation studies, less attention has been given to understanding the psychological factors that influence the formation of attitude in individuals as main determinant of intention. There is also a research gap concerning the Zagros region, an area of significant forest importance. Although numerous conservation programs have been designed and implemented in this region over the years, they have not achieved the desired success, primarily due to the lack of public participation. Examining and identifying the determinants of individuals’ attitudes represents a crucial and initial step in the development of participatory programs. By identifying the factors that shape attitudes, educational programs can be designed to strengthen positive attitudes toward forest conservation and influence individuals’ behavior. This study aims to address this gap by identifying the factors that influence the formation of individuals’ attitude toward forest conservation. In this research variables from VBN and NAM were integrated into a model to explore their influence on conservative attitude of individuals toward forest conservation.

## 2 Conceptual framework of study

**Figure 1:**
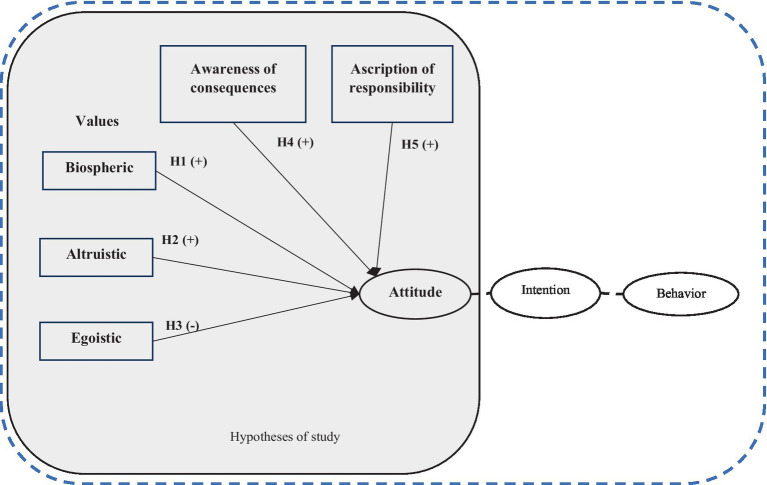
Conceptual framework of study.

The conceptual model of this study is illustrated in Figure 1. As illustrated in the model, attitude is considered the dependent variable, with the influence of independent variables such as values, awareness of consequences, and ascription of responsibility being examined. Attitudes refer to individuals’ evaluations of a behavior, reflecting the extent to which they perceive it favorably or unfavorably (Ajzen, 2011). Positive attitudes toward pro-environmental behaviors are associated with greater intention to engage in such actions. The significance of attitudes in shaping behavior lies in their role as the primary determinant of individuals’ behavioral intention, which, under certain conditions, can lead to actual behavior. In studies focused on forest conservation, it has been established that attitudes exert a strong and significant impact on individuals’ behavioral intention (Maleknia et al., 2024; Mohammadi et al., 2024a). The individuals’ environmental behaviors are significantly influenced by their underlying values, beliefs about environmental issues, and the social norms within their communities (Lind et al., 2015). In the environmental context, these values include ideals such as ecological preservation, sustainability, and respect for nature. Biospheric values, for example, relate to the well-being and preservation of non-human species and the broader biosphere. Individuals with biospheric values prioritize the health of ecosystems, biodiversity, and the natural environment (Stern et al., 1999). Conversely, altruistic values pertain to individuals who are primarily concerned with the welfare and well-being of other people, motivated by empathy, compassion, and a sense of social responsibility (Stern et al., 1995). Egoistic values, on the other hand, revolve around self-interest and personal gain. Individuals with egoistic values prioritize their own well-being, social status, power, control over others, and accumulation of wealth. Awareness of consequences is a critical component of the VBN theory which refers to the understanding of the negative impacts that one’s actions can have on the environment and is essential for fostering a sense of responsibility toward pro-environmental behavior. Ascription of responsibility refers to the extent to which individuals attribute accountability for environmental problems to themselves or others, influencing their environmental behavior (Ai and Rosenthal, 2024; Stern et al., 1999). When individuals feel personally responsible for environmental issues, they are more likely to engage in pro-environmental actions. This concept is central to environmental psychology and is often linked to moral obligation and personal norms which suggests that when people perceive responsibility for environmental degradation, they develop stronger personal norms to act in environmentally friendly ways (Oh and Ki, 2022).

Studying the influences of these variables on attitude offers several conceptual and empirical advantages in understanding pro-environmental attitude. Firstly, these variables provide a comprehensive base for conceptualizing the antecedents of environmental attitude (Stern et al., 1995), which is a central component of intention (Harland et al., 1999). Limited studies have examined the impact of these factors on individuals’ attitudes. For example, Al Mamun et al. (2022) and Kim et al. (2016) confirmed the role of these constructs on individuals’ attitudes toward environmental behaviors. However, in the field of forest conservation, studies have focused on examining the impact of attitudes on individuals’ behavioral intentions within the TPB model. The innovation of this study lies in integrating these constructs within the context of forest conservation. Specifically, this study aims to examine the influence of values from VBN model and awareness of consequences and ascription of responsibility from NAM on individuals’ conservation attitudes, which serve as a significant determinant of behavioral intention. By incorporating these constructs into a model to explore the shaping factors of intention, researchers can capture the broader socio-psychological context in which attitudes are formed. Secondly, these independent variables which are from VBN and NAM theory highlights the significance of intrinsic motivations and moral considerations in driving pro-environmental behavior, which may not be fully accounted for by the behavioral models’ emphasis on rational decision-making processes. Integrating constructs from VBN and NAM theories into the psychological framework with a construct of attitude enables researchers to better understand the role of moral imperatives and social identity in shaping environmental attitudes. Thirdly, from a research perspective, this framework provides a theoretical basis for exploring the mechanisms through which selected variables influence attitudes, facilitating the development of more effective interventions and policies. From a practical standpoint, this model can inform the design of interventions aimed at promoting sustainable behaviors and lifestyles. By understanding the underlying variables that influence individuals’ environmental attitudes, practitioners can tailor communication strategies and behavior change interventions to resonate with target audiences and foster positive environmental behaviors. Accordingly, the research hypotheses were formulated as follows:

*H1*. Biospheric values positively influence individuals’ attitudes.

*H2*. Altruistic values positively influence individuals’ attitudes.

*H3*. Egoistic values have a negative and significant impact on individuals’ attitudes.

*H4*. Awareness of consequences positively influences individuals’ attitudes.

*H5*. Ascription of responsibility positively influences individuals’ attitudes.

The research hypotheses are illustrated in the conceptual model of the research, as shown in Figure 1.

## 3 Materials and methods

### 3.1 Study area

This research study was conducted at the Faculty of Natural Resources at Behbahan University as a case study, situated in the city of Behbahan in the southwestern region of Iran. Notably, this city is located in close proximity to the Zagros forests, which encompass a vast expanse of approximately 5 million hectares and represent one of Iran’s most vital natural habitats. The Zagros forests play a pivotal role in various ecological aspects, including water provisioning, soil conservation, and the sustenance of local communities (Khosravi et al., 2016; Rashidi et al., 2024). In recent decades, these invaluable forest ecosystems have faced severe degradation due to multiple factors, such as extensive wildfires (Savadroodbari et al., 2017), land-use changes (Parma et al., 2017), unregulated grazing practices (Khezri et al., 2017), and unsustainable exploitation by the local populace (Mahmoudi et al., 2023). There is also a forest degradation due to local communities’ dependence on forests for energy (Bazgir et al., 2024) and high pressure of tourists (Latifinia et al., 2022). Despite their immense ecological significance, the Zagros forests have been subjected to substantial destruction, necessitating urgent and effective conservation measures. Consequently, safeguarding and preserving these forests have emerged as critical challenges that demand immediate attention and concerted efforts. However, numerous efforts to conserve these forests have not been successful. One of the primary reasons for these failures is the lack of public participation in conservation programs (Khedrizadeh et al., 2017; Khosravi et al., 2014). The present study, conducted within the context of Behbahan University, offers valuable insights into the attitudes and perceptions of students majoring in Natural Resources toward environmental conservation.

### 3.2 Population of study and sampling method

This study was conducted among students studying in Natural Resources at Behbahan University in Khuzestan Province, Iran. The reason for selecting this population for the research was to eliminate the influence of certain other factors. For example, demographic characteristics such as age, education level, or income can have an impact on individuals’ attitudes. Therefore, a homogenous population was selected in terms of these characteristics to control for the mentioned variables and minimize their effect on differences in individuals’ attitudes. The target population consisted of all students of Natural Resources at this university, which amounted to 264 individuals. Based on Krejcie and Morgan (1970) a total number of 165 samples is necessary for this population. For more precision, a sample size of 200 was sampled for study. The major age range of the participants was 20–30 years, and 55% of the participants were male students, while 45% were female students. The sampling was conducted randomly among the students. Sufficient explanations about the research were provided to all participants. Consent was obtained from all individuals to use the information provided by them. They were assured that the information presented would be used solely for the purpose of this study.

### 3.3 Data collection

The data for this research study was collected using a questionnaire designed to investigate individuals’ perspectives on various constructs. The questionnaire consisted of several sections. The first section was designed to cover independent constructs of study which are presented in Table 1. These constructs included values, which encompassed three categories: biospheric, altruistic and egoistic. Additionally, two variables, ascription of responsibility, and awareness of consequences, were included. In the second section, the construct of attitude was assessed as the dependent variable. Prior to data collection, the questionnaire was reviewed by a panel of experts consisting of 9 persons in different disciplinaries including in forestry, extension and education, environmental science, and watershed management to ensure its content validity. The questionnaire was revised based on comments from panel members and the revised version was confirmed by panel. Subsequently, a pilot test was conducted using the questionnaire, and a sample of 30 participants from students at university was selected. The results of this pilot test indicated that all the questionnaire variables exhibited Cronbach’s alpha coefficients exceeding 0.85, indicating high reliability. The questionnaires were distributed among participants face to face. They were provided with the necessary information regarding the study and enough time to complete questionnaires.

**Table 1:** The variables and statements of study with reliability and validity test results.

Variables	Code	Statements	VIF	Reliability and validity	References
Biospheric values	BV1	I prioritize conserving forests for future generations	2.093	α = 0.881 CR = 0.926 AVE = 0.808	Latifinia et al. (2022)
	BV2	The conserving forest ecosystems is value for me	1.087		
	BV3	I value the interconnectedness of forests and ecological balance	1.374		
Altruistic values	AV1	I prioritize forest conservation for the benefit of society	2.438	α = 0.817 CR = 0.890 AVE = 0.730	Ghazali et al. (2019)
	AV2	I support initiatives for the well-being of forests	2.948		
	AV3	I think I have to sacrifice my time for the health of forest ecosystems	2.854		
Egoistic values	EV1	My personal interests come first in forest conservation	1.391	α = 0.812 CR = 0.889 AVE = 0.727	Hong et al. (2024)
	EV2	I prioritize immediate gains over long-term forest sustainability	2.683		
	EV3	I focus on personal benefits rather than broader impacts on forests	2.570		
Awareness of consequences	AC1	I consider the impacts of forest actions on ecology, society, and the economy	2.587	α = 0.852 CR = 0.909 AVE = 0.770	Latifinia et al. (2022)
	AC2	I stay informed about the consequences of forest practices	2.488		
	AC3	I’m aware of how human actions affect forests	2.688		
Ascription of responsibility	AR1	I have a duty to actively participate in forest conservation	2.653	α = 0.907 CR = 0.942 AVE = 0.843	Zhang et al. (2020)
	AR2	I take responsibility for promoting sustainable forest practices	1.087		
	AR3	I feel accountable for the well-being of forests	1.052		
Attitude	ATT1	Conserving forests is important to me	1.350	α = 0.899 CR = 0.937 AVE = 0.832	Mohammadi et al. (2024a) and Savari and Khaleghi (2023a)
	ATT2	Conserving forest in necessary	2.120		
	ATT3	Conserving forest is wise measure			

### 3.4 Data analysis

In this research, Structural Equation Modeling (SEM) was applied to analyze the influence of constructs on attitude. Specifically, Partial Least Squares Structural Equation Modeling (PLS-SEM) was utilized, a powerful method ideal for addressing complex models, especially when working with smaller sample sizes or non-normally distributed data. SEM is divided into two key components: the measurement model, which captures the links between observed indicators and their latent variables, and the structural model, which investigates the connections between the latent variables (Shahangian et al., 2024). PLS-SEM offers several advantages, such as the ability to accommodate both reflective and formative measurement models, as well as its flexibility when dealing with less strict data requirements compared to covariance-based SEM (Hair and Alamer, 2022). Additionally, it is particularly effective in exploratory research since it focuses on maximizing the explained variance of dependent variables, making it a valuable tool for identifying significant factors driving behavior in this study (Hair et al., 2019).

To ensure the reliability of the measurement items pertaining to the constructs within the proposed model, statistical measures such as Cronbach’s alpha coefficient (Cronbach, 1951), Average Variance Extracted (AVE), and Composite Reliability (CR) were computed. The AVE is determined by averaging the squared factor loadings of a construct’s indicators, showing how much variance is captured by the construct compared to the total variance. A value above 0.50 suggests that the construct accounts for more than half of the variance in its indicators (Hair and Alamer, 2022). CR is calculated by dividing the squared sum of the factor loadings by the sum of squared loadings and error variances. It measures internal consistency, with values greater than 0.70 indicating acceptable reliability (Hair et al., 2019). Additionally, discriminant validity was addressed, which involves ensuring that a construct is truly distinct from other constructs. This can be evaluated using various criteria such as the Fornell-Larcker and Heterotrait-Monotrait Ratio (HTMT) The Fornell-Larcker criterion assesses discriminant validity by comparing the square root of the AVE for each construct to its correlations with other constructs. Discriminant validity is considered adequate when the square root of the AVE for a construct exceeds its correlations with any other construct (Fornell and Larcker, 1981). The HTMT is another approach to evaluating discriminant validity, which examines the relationships between constructs. For discriminant validity to be established using HTMT, the ratio should fall below a specified threshold, typically 0.85 or 0.90 (Dijkstra and Henseler, 2015). In this study, Confirmatory Factor Analysis (CFA) was employed to assess the congruence between the observed data and a pre-established conceptual model. CFA serves as a framework for outlining the anticipated relationships among latent factors and their observed indicators. Its application enables researchers to examine the degree to which the observed data align with the hypothesized model (Xing et al., 2023). The determination of factor loadings for all constructs within the model was carried out to evaluate the unidimensional nature of the chosen variables in the measurement models. To establish strong evidence supporting the unidimensionality of the variables, it is recommended that the factor loadings surpass the threshold of 0.5 (Hair et al., 2017). This criterion signifies that each observed indicator variable should exhibit a robust association with its corresponding latent construct. Variance Inflation Factor (VIF) was used to assess multicollinearity in the model. VIF values indicate the extent to which multicollinearity is present among the independent variables (Shahangian et al., 2024). Data analysis for this research study involved the utilization Smart-PLS3_3_ Software to assess the reliability and validity of the collected data, as well as to conduct path analysis.

## 4 Results

### 4.1 Results of validity and reliability

Table 1 illustrates the variables of the research model, and the corresponding statements used to measure these variables. In this table, Cronbach’s alpha, AVE, and CR are reported for each of the research variables. The Cronbach’s alpha coefficients ranging from 0.812 to 0.907 indicate strong internal consistency, providing evidence of high reliability in the survey instrument (Cronbach, 1951). As shown in the table, all constructs in the research model exhibited a CR value above 0.890 (Hair et al., 2019; Shahangian et al., 2024). Moreover, the AVE values for all constructs exceeded the threshold of 0.50. These findings collectively support the satisfactory reliability and validity of the latent variables in the model (Fornell and Larcker, 1981). Consequently, it can be inferred that the selected items effectively measure the constructs and were carefully chosen. The square root of the AVE for the research constructs, ranging from 0.853 to 0.918, was observed to be higher than the correlations between them, which ranged from −0.331 to 0.768 (Table 2). These findings provide evidence of discriminant validity among the constructs in the proposed research model (Fornell and Larcker, 1981). In other words, the constructs are distinct from one another and do not overlap significantly in terms of their measurement. This supports the notion that each construct measures a unique aspect of the phenomenon under investigation. In Table 3, the values represent the HTMT ratios between the constructs. The ratios fall well below the threshold of.85, further confirming that these constructs are adequately distinct from one another (Dijkstra and Henseler, 2015). The results showed that all VIF values were below the commonly accepted threshold of 3, indicating that multicollinearity is not a concern, and the predictors are sufficiently independent of one another for reliable regression analysis (Hair et al., 2019).

**Table 2:** The results of Fornell and Larcker of variables of study.

Constructs	AV	AR	Attitude	AS	BV	EV
Altruistic values	0.855					
Ascription of responsibility	0.664	0.918				
Attitude	0.710	0.704	0.912			
Awareness of consequences_	0.750	0.598	0.768	0.878		
Biospheric values	0.645	0.519	0.678	0.626	0.899	
Egoistic values	−0.229	−0.291	−0.331	−0.275	−0.249	0.853

**Table 3:** The results of HTMT criterion.

Constructs	AV	AR	Attitude	AS	BV
Altruistic values					
Ascription of responsibility	0.451				
Attitude	0.291	0.319			
Awareness of consequences_	0.761	0.497	0.387		
Biospheric values	0.659	0.488	0.278	0.841	
Egoistic values	0.58	0.365	0.328	0.780	0.611

### 4.2 Path analysis and hypothesis results

**Figure 2:**
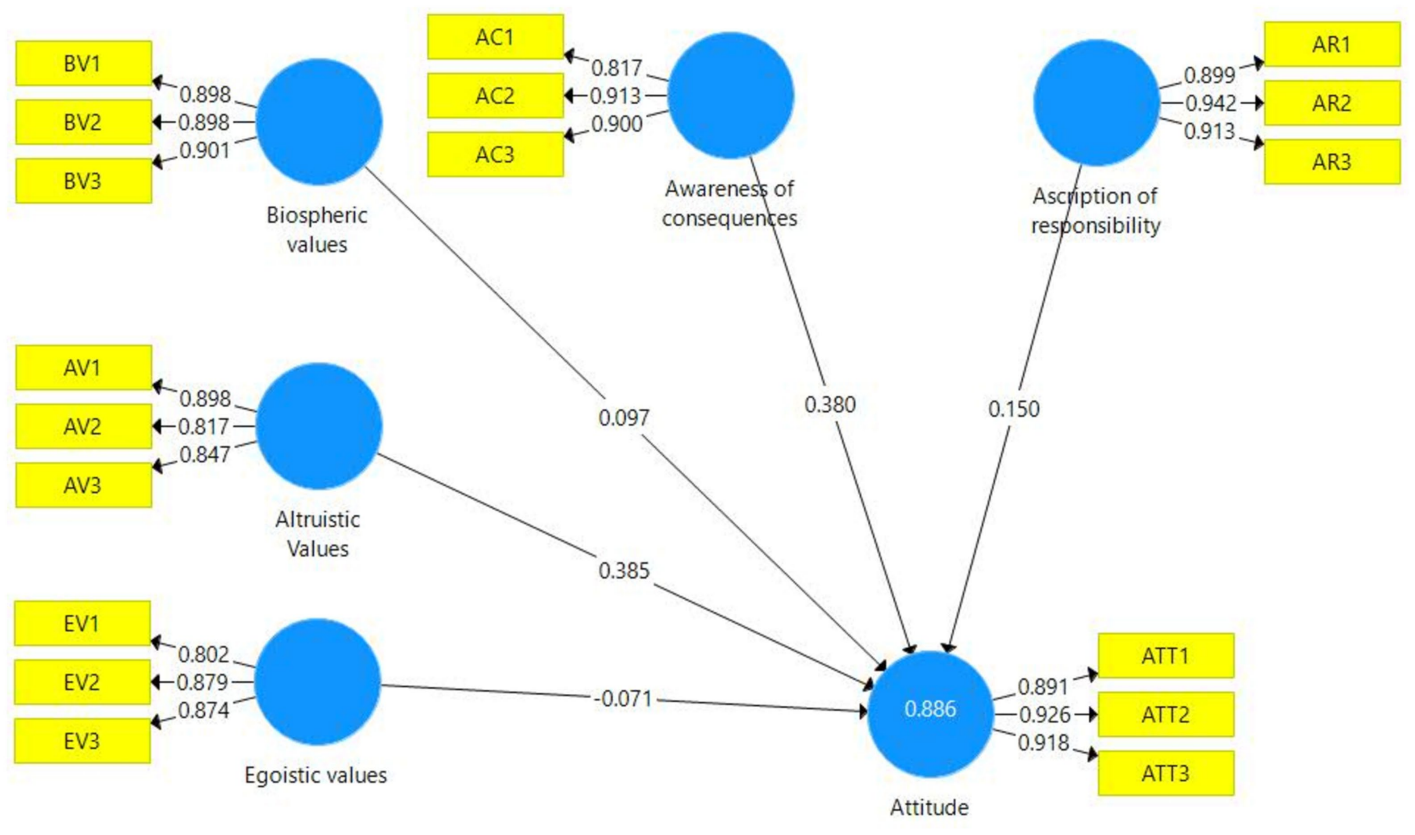
The structural model of study with path coefficients.

In Figure 2, the path analysis for the conceptual model of the research is presented. The results of hypotheses test are also presented in Table 4. As evident from the figure, the factor loadings for all items used in the study exceed 0.800, indicating appropriate item selection for measuring the model variables (Hair et al., 2019). The results demonstrate that the selected variables for the specific research model are capable of explaining 88.6% of the variance in individuals’ attitudes toward forest conservation. The path coefficients indicate that altruistic values (0.385), awareness of consequences (0.380), ascription od responsibility (0.150) and biospheric values (0.097) have the greatest impact on individuals’ attitudes, respectively. Furthermore, egoistic values have a negative influence (−0.071) on individuals’ attitudes toward forest conservation. The results of hypotheses test confirmed the hypothese 1. The hypothes 1 explored the influence of biospheric values on individuals’ attitudes. The hypothesis test revealed a statistically significant positive influence of biospheric values on attitude (*t* = 3.637, *p* = 0.00) suggesting that individuals who prioritize environmental concerns and tend to hold more positive attitudes. The hypothesis test for H2 demonstrated a significant positive influence of altruistic values onattitude (*t* = 7.268, *p* = 0.000). This finding suggests that individuals who value selflessness, empathy, and the welfare of others display more favorable attitudes. The reulst of test for H3 yielded a statistically significant but negative influence of egoistic values on attitude (*t* = 3.484, *p* = 0.001). It indicates that individuals who prioritize self-interest and personal gain tend to exhibit more negative attitude toward forest conservation. The hypothesis test demonstrated a highly significant relationship between awareness of consequences and attitude (H4) (*t* = 8.164, *p* = 0). This result suggests that individuals who possess a heightened awareness of the potential outcomes and impacts of their actions tend to hold more favorable attitudes. Hypothesis 5 tsted the influence of scription of responsibility on attitude. The hypothesis test revealed a statistically significant and positive influence of ascription of responsibility on attitude (*t* = 5.143, *p* = 0.000). This finding suggests that individuals who attribute responsibility to themselves for environmental problems and issues probably have more positive attitudes. Based on the results presented in Table 3, it can be concluded that all the variables in the study had a significant effect in this section. According to the factor loadings and *f*^2^ values, the variables altruistic values and awareness of consequences had the most substantial impact on the attitude variable. Furthermore, considering that the *Q*^2^ value for the endogenous constructs in the model was greater than zero, it can be stated that the formulated path model has predictive capability for the structural relationships. In other words, the path model demonstrates good predictive fit with the endogenous constructs (Hair et al., 2019).

**Table 4:** The results of hypotheses test.

		*f* ^2^	*R* ^2^	*Q* ^2^	Bias corrected confidence interval		T statistics	*p* values	Result
H1	Biospheric values → Attitude	0.04	0.886	0.576	0.151	0.341	3.637	0.000^**^	Confirmed
H2	Altruistic values → Attitude	0.17			−0.007	0.118	7.268	0.000^**^	Confirmed
H3	Egoistic values → Attitude	0.03			−0.116	−0.012	3.484	0.001^**^	Confirmed
H4	Awareness of consequences → Attitude	0.16			0.305	0.532	8.164	0.000^**^	Confirmed
H5	Ascription of responsibility → Attitude	0.09			0.237	0.398	5.143	0.000^**^	Confirmed

**Significant at the 0.01 level.

These findings suggest that increasing individuals’ awareness of the consequences of forest conservation, strengthening altruistic values, fostering a sense of responsibility alongside environmental values, can enhance individuals’ attitudes toward forest conservation. Additionally, egoistic attitudes in individuals weakens their attitudes toward forest conservation.

## 5 Discussion

The present study aimed to examine the factors shaping individuals’ attitudes toward forest conservation. To this end, the influence of individuals’ values from VBN model and awareness of consequences and ascription of responsibility from theory of NAMon their attitudes was investigated. The findings of the study revealed that the variables considered in specific model of study were able to explain 88.6% of individuals’ attitudes toward forest conservation. This result clearly indicates that these variables significantly shape individuals’ attitudes. Other studies have also shown that individuals’ values, awareness of consequences and ascription of responsibility can influence on their attitudes toward environmental behaviors (Al Mamun et al., 2022; Kim et al., 2016; Savari et al., 2023).

The hypothetical test results indicated that biospheric values have a significant impact on individuals’ attitudes. Other studies have also demonstrated that biospheric values or values related to environmental issues are predictive factors of individuals’ attitude toward environmental behavior (Bouman et al., 2020; Joffe-Nelson et al., 2024; Wang et al., 2021). Therefore, enhancing individuals’ environmental knowledge and awareness regarding the importance of forest conservation can bolster their biospheric values (Latifinia et al., 2022), subsequently fostering a positive attitude toward the conservation of these resources. The individuals with robust biospheric values recognize the intrinsic value of forests, appreciating their ecological functions, biodiversity, and the ecosystem services they provide (Ihemezie et al., 2021). As a result, they are more likely to adopt pro-conservation attitudes and engage in behaviors that support forest conservation efforts. They may actively participate in reforestation projects, advocate for stricter regulations against deforestation, or support organizations working toward forest conservation (Zhang et al., 2022). Their attitudes are often driven by a sense of responsibility toward future generations and a desire to maintain the integrity of the natural environment.

The hypothesis results confirmed a significant and positive impact of altruistic values on individuals’ attitude toward forests conservation. Altruistic values encompass a concern for the welfare and interests of others, extending beyond one’s personal well-being. Although no research explored the direct influence of this variable on individuals’ attitude toward forests conservation, research showed altruistic individuals tend to prioritize the welfare of others, fairness, and environmental conservation over personal gains (Druica et al., 2023; Sánchez et al., 2018; Wang and Udall, 2023). They are more likely to engage in sustainable behaviors such as forest conservation (Latifinia et al., 2022). Research findings have demonstrated that these values can contribute to improving individuals’ environmental awareness (Momenpour et al., 2024). Individuals with strong altruistic values exhibit empathy and compassion toward other human beings, as well as the broader ecosystem. They recognize the societal benefits derived from forests, such as clean air, water regulation, and climate regulation, and they prioritize the conservation of forest. Individuals with strong altruistic values are more likely to perceive forests as a common heritage and recognize the importance of their preservation for the benefit of present and future generations. They often engage in pro-conservation behaviors such as supporting sustainable forest management practices, advocating for conservation policies, and participating in community-based conservation initiatives. This influence can be related to influence of this value on attitude. It was showed in this study that the attitude can influenced by this variable positively and this impact can translate to intention and behavior indirectly.

The results revealed the significant negative of egoistic values on participants’ attitudes toward forest conservation. Individuals with strong egoistic values prioritize their immediate needs and desires over broader environmental concerns (Stern et al., 1999). Although the influence of this variable on individuals’ attitude toward forest conservation was not explored, research showed that egoistic values can negative pro-environmental intention of people (De Groot and Steg, 2010; Tamar et al., 2020) The individuals with higher rate of egoistic values may view forests primarily as a source of economic opportunity or personal benefit, such as timber extraction or land development, without considering the long-term consequences of such actions (Mohammadi et al., 2024b). They may perceive conservation efforts as hindrances to economic growth or personal gain, leading to resistance or opposition to conservation initiatives. Egoistic values often prioritize short-term benefits over long-term sustainability, which can undermine forest conservation efforts and contribute to deforestation and habitat destruction. It is necessary, therefore, for promotional educational programs to outline the public benefits of forest conservation and concurrently reinforce individuals’ altruistic and biospheric values. This will discourage individuals from sacrificing forest protection for their personal needs.

Research findings have demonstrated that awareness of the consequences has a positive and significant impact on individuals’ attitudes toward forest conservation. Research mostly explored the impact of this variable on individuals’ intention (Badawi et al., 2024; Lv et al., 2021), but study have indicated that awareness of consequences influences the attitudes of rural women toward environmental issues (Karimi and Mohammadimehr, 2022). Awareness of consequences can strengthen individuals’ environmental understanding of the necessity of certain behaviors and motivate them to engage in those behaviors (Fang et al., 2020). Therefore, it can be asserted that increasing individuals’ awareness regarding the importance of forest conservation enhances their attitudes, which subsequently influences their behavioral intentions and, ultimately, their actual behavior toward forest conservation. Awareness-raising programs play a vital role in cultivating such attitudes among individuals.

The results of the study indicate that the ascription of responsibility has a positive impact on individuals’ attitudes toward forest conservation. In the environmental-related studies, ascription of responsibility has been recognized as a significant factor influencing pro-environmental behavior (Al Mamun et al., 2022; Bøhlerengen and Wiium, 2022; Chwialkowska et al., 2020). When individuals perceive a sense of personal responsibility for environmental issues, they are more likely to engage in behaviors that contribute to environmental protection (Pawaskar et al., 2020). The ascription of responsibility entails attributing oneself as a causal agent in environmental matters, acknowledging the role and impact of personal actions on the environment. This sense of responsibility creates a cognitive connection between individuals and the environment, leading to increased awareness and concern for environmental issues (Latifinia et al., 2022) which can shape conservative attitude about forests. As individuals’ sense of responsibility increases, their attitude toward forest conservation is probably strengthened, leading them to engage in forest conservation behaviors. Moreover, it can serve as a foundation for individuals’ intention to participate in forest conservation programs.

This study had certain limitations. Firstly, it focused on examining the impact of factors on individuals’ attitudes, without investigating the influence of these variables on individuals’ behavioral intentions. Therefore, exploring the direct and indirect effects of these factors on individuals’ behavioral intentions can provide a better understanding of their effects. Moreover, this study was conducted in a specific community with distinct characteristics and within a specific geographical area. Caution should be exercised when generalizing the results. This approach stemmed from the study’s intention to control for other variables influencing attitudes by using a homogeneous sample in terms of age, education, occupation, and income. Therefore, it is essential to replicate this study with a diverse sample population in terms of demographic characteristics in order to gain a comprehensive understanding of the determinants of individuals’ attitudes. Additionally, conducting this study in other regions would contribute to a better understanding of the effects of these variables on attitudes. The results of this study can be utilized in promotional and educational programs to enhance individuals’ conservation attitudes toward forest conservation. Considering that 88.6% of the variance in individuals’ attitudes toward forest conservation was explained, future research can incorporate additional variables into the conceptual framework or integrate components of other behavioral models with this conceptual framework to explain a larger portion of the variance in individuals’ attitudes.

## 6 Theoretical and practical implications

This study has both theoretical and practical implications that can be considered in future research or implementation programs. The present study was able to explain 86.6% of the variance in individuals’ attitudes toward forest conservation. Therefore, this study establishes a framework for understanding the determinants of individuals’ attitudes toward forest conservation. Although conducted in the Zagros forests, this model can be applied to assess attitudes in other regions as well. The present study highlights the significance of values, awareness of consequences, and ascription to responsibility in shaping individuals’ attitudes. In doing so, it sheds light on the previously unexplored role of these factors in shaping attitudes toward forest conservation. While several previous studies have aimed to understand behavioral intentions or environmental behaviors in general contexts or specifically in forests, this study offers a novel approach by integrating components from the VBN and the NAM, explaining a substantial portion of the variance in attitudes toward forest conservation. These findings can inform future research and conservation planning efforts. Strengthening conservation attitudes by considering the factors identified in this research may lead to enhanced behavioral intentions and actual behaviors. Additionally, an important aspect of this research is its focus on the forest sector, where environmental behavior studies are relatively scarce. In forests where there are complex socio-economic conditions and local livelihoods depend heavily on forest resources, this research gap is particularly evident. Hence, this study takes an initial step toward addressing this issue and paves the way for further research. Understanding these dynamics can contribute to the design of complex participatory conservation programs in similar forest settings.

This study has also implications for policy-making. Given the positive influence of biospheric values and awareness of consequences, it is necessary to develop awareness-raising programs to enhance individuals’ environmental knowledge and the awareness of outcomes of their conservation actions. This can increase the probability of actual behavior change in individuals. On the other hand, educational and promotional cultural programs can strengthen values of altruism and ascription of responsibility in individuals while reducing egoistic values. These programs can enhance individuals’ conservation attitudes, thereby increasing the chance of engaging in conservation behaviors. Additionally, fostering a sense of responsibility among individuals for forest conservation, educating them about the consequences of forest degradation, and their non-participation in conservation efforts can lead to a strengthened protective attitude. Providing the conditions and opportunities to transform this attitude into behavioral intention and engaging the public to convert this intention into actual behavior can facilitate forest conservation by garnering public participation. Given that the lack of public participation in forest conservation programs is one of the primary reasons for the failure of these programs in this region, it is necessary to design participatory programs based on the reinforcement of individuals’ attitudes, in light of the results of this study.

## 7 Conclusion

The present study aimed to examine the determinants of individuals’ attitude toward forest conservation. The influence of variables associated with values from VBN theory, awareness of consequences, and ascription of responsibility from NAM on individuals’ attitudes toward forest conservation was explored. The results indicated that biospheric and altruistic values, ascription of responsibility, and awareness of consequences have significant and positive effects on individuals’ conservation attitudes. However, egoistic values have a negative impact on individuals’ conservation attitudes. The research findings hold significant implications for researcher and policymakers involved in forest conservation efforts. By understanding the factors that shape individuals’ conservation attitudes, decision-makers and researchers can develop targeted strategies, research and interventions to strengthen positive attitudes toward forest conservation.

## Data Availability

The raw data supporting the conclusions of this article will be made available by the authors, without undue reservation.
